# *Vibrio parahaemolyticus* bacteremia in decompensated cirrhosis complicated by spontaneous bacterial peritonitis following occupational seafood exposure: A case report

**DOI:** 10.1016/j.idcr.2026.e02802

**Published:** 2026-10-14

**Authors:** Thi Vy Ngoc Truong, Hac-Tu T. Chung, Sai Pavan Kumar Chintalapati, Pooja Gurram

**Affiliations:** a College of Medicine, University of Florida, Gainesville, FL, USA; b Department of Infectious Diseases, College of Medicine, University of Florida, Gainesville, FL, USA

**Keywords:** Vibrio parahaemolyticus, Bacteremia, Cirrhosis, Spontaneous bacterial peritonitis, Occupational exposure, Case report

## Abstract

**Introduction:**

*Vibrio parahaemolyticus* is a halophilic marine Gram-negative bacterium most commonly associated with seafood-related gastroenteritis. Invasive infection is uncommon but may be severe in patients with chronic liver disease, and non-ingestive occupational exposure is an underrecognized route of infection.

**Presentation of case:**

A man in his early 60 s with alcohol-associated decompensated cirrhosis and recurrent ascites presented with abdominal pain and sepsis. Ascitic fluid contained > 10,000 polymorphonuclear cells/µL, meeting diagnostic criteria for spontaneous bacterial peritonitis (SBP), although ascitic cultures remained negative. Four of four blood-culture bottles grew *V. parahaemolyticus*. He denied raw seafood ingestion but had worked as a fish cleaner for two years with frequent minor skin lacerations. Bacteremia cleared and ascitic neutrophilia improved with cephalosporin and doxycycline therapy, but his course was complicated by progressive multiorgan failure and subsequent *Candida glabrata* fungemia. He died after transition to comfort-focused care.

**Discussion:**

The concurrent bacteremia and marked culture-negative neutrocytic ascites raise the possibility of hematogenous peritoneal involvement, but organism-specific SBP could not be microbiologically confirmed. Occupational inoculation through disrupted skin was considered a plausible portal of entry.

**Conclusion:**

In patients with cirrhosis and sepsis, clinicians should assess occupational and environmental marine exposures even when seafood ingestion is absent and should avoid attributing culture-negative SBP to a bloodstream isolate without direct peritoneal microbiologic confirmation.

## Introduction

*Vibrio parahaemolyticus* is a halophilic Gram-negative bacterium and an important cause of seafood-associated gastroenteritis worldwide [1], [2], [3], [4]. In the United States, infections are particularly associated with coastal regions and warmer months [4], [5]. Most infections are self-limited; however, invasive disease, including wound infection, bacteremia, septicemia, and necrotizing soft-tissue infection, can occur [3], [6], [7], [8], [9], [10].

Severe *V. parahaemolyticus* infection is more likely in patients with chronic liver disease, cirrhosis, diabetes mellitus, immunosuppression, and other major comorbidities [6], [7], [8]. Infection most commonly follows ingestion of raw or undercooked seafood, particularly shellfish, but non-ingestive transmission through contaminated seawater, brackish water, market water, or occupational seafood exposure has also been described [7], [9], [10], [11], [12], [13].

Spontaneous bacterial peritonitis (SBP) is a common and potentially life-threatening complication of decompensated cirrhosis. Typical organisms include enteric Gram-negative bacteria such as Escherichia coli and Klebsiella pneumoniae [14], [15]. *V. parahaemolyticus* is rarely reported in association with neutrocytic ascites or SBP. We report confirmed *V. parahaemolyticus* bacteremia occurring concurrently with culture-negative neutrocytic ascites meeting criteria for SBP in a patient with advanced cirrhosis and recurrent occupational raw-fish exposure through minor skin trauma.

## Case presentation

A man in his early 60 s with alcohol-associated decompensated cirrhosis and recurrent tense ascites requiring approximately twice-weekly therapeutic paracentesis presented to a referring hospital with progressive abdominal distension and worsening abdominal pain. Initial laboratory testing showed a white blood cell count of 17 × 10⁹/L, lactate 2.7 mmol/L, total bilirubin 8.2 mg/dL, INR 2.1, and serum creatinine 2.3 mg/dL, subsequently increasing to 4.04 mg/dL from a baseline of approximately 1.1 mg/dL.

Diagnostic paracentesis demonstrated markedly neutrocytic ascites, with polymorphonuclear cells comprising 82% of the ascitic leukocyte population and an absolute neutrophil count > 10,000 cells/µL, meeting diagnostic criteria for SBP. Ascitic fluid culture showed no growth. Blood cultures obtained at presentation demonstrated Gram-negative bacilli in all four bottles. An initial rapid molecular blood-culture identification panel did not identify an organism. Approximately two days later, conventional cultures identified *Vibrio parahaemolyticus* in all four bottles.

The patient was initially treated with ceftazidime. Antimicrobial therapy was subsequently broadened to cefepime, doxycycline, and metronidazole as the microbiologic diagnosis evolved. His acute kidney injury worsened and required intermittent hemodialysis. He was transferred to our tertiary-care center for management of advanced liver disease and evaluation for liver transplantation.

On transfer, he denied consumption of raw or undercooked seafood and reported a shellfish allergy. Additional history revealed that he lived in coastal Florida and had worked as a fish cleaner for approximately two years. His occupation involved frequent handling of raw fish and recurrent minor skin lacerations. He denied recent diarrhea or rash, and no cellulitis, bullae, necrosis, or active soft-tissue infection was identified on examination.

At our institution, antimicrobial therapy was subsequently narrowed to ceftriaxone 2 g intravenously every 24 h plus doxycycline 100 mg intravenously every 12 h based on the microbiologic diagnosis and susceptibility information. Follow-up blood cultures remained negative. Repeat paracentesis demonstrated a substantial decline in the ascitic neutrophil count, although ascitic cultures remained negative. Prior antimicrobial exposure may have reduced culture yield.

Secondary peritonitis was considered because of the marked ascitic neutrophilia, abdominal symptoms, and subsequent bowel dysfunction. However, available imaging did not demonstrate gastrointestinal perforation or another surgically correctable intra-abdominal source. The patient therefore met clinical criteria for culture-negative neutrocytic ascites/SBP, but direct microbiologic confirmation of *V. parahaemolyticus* from the peritoneal fluid was not obtained.

Despite microbiologic clearance of bacteremia, the patient developed worsening distributive shock requiring norepinephrine and vasopressin and progressive renal failure requiring continuous renal replacement therapy. Recurrent tense ascites required repeated therapeutic paracenteses. His course was further complicated by ileus and small-bowel obstruction requiring nasogastric decompression and total parenteral nutrition, as well as severe coagulopathy requiring blood-product support.

Later in the hospitalization, blood cultures grew *Candida glabrata*. A central venous catheter-associated source was suspected, and micafungin was initiated. The patient underwent formal liver-transplant evaluation but was ultimately not considered a transplant candidate. Despite maximal supportive care, he continued to have progressive hemodynamic and multiorgan failure. Following multidisciplinary discussions with his surrogate decision-maker, care was transitioned to comfort-focused measures. He died 16 days after transfer.

## Discussion

*Vibrio parahaemolyticus* is predominantly associated with gastroenteritis after ingestion of raw or undercooked seafood. Invasive infection is substantially less common but can result in bacteremia, septic shock, necrotizing soft-tissue infection, and death, particularly in patients with chronic liver disease and other significant comorbidities [3], [6], [7], [8], [9], [10].

This case has two notable features. First, invasive *V. parahaemolyticus* infection occurred without reported seafood ingestion. Second, bacteremia occurred in temporal association with marked neutrocytic ascites in a patient with advanced cirrhosis. The latter finding raises the possibility of Vibrio-associated peritoneal involvement but does not establish it microbiologically.

Non-ingestive and invasive *V. parahaemolyticus* infections are uncommon compared with gastroenteritis but can cause severe disease, particularly in susceptible hosts. Dechet et al. demonstrated substantial morbidity and mortality from nonfoodborne Vibrio infections in the United States, particularly among patients with underlying liver disease [7]. Published cases of severe *V. parahaemolyticus* infection demonstrate a broad clinical spectrum, ranging from cellulitis and necrotizing soft-tissue infection to bacteremia, septicemia, septic shock, and multiorgan failure (Table 1) [8], [9], [11], [12], [13], [16], [17], [18].

**Table 1:** Clinical characteristics, exposures, and outcomes of published severe and invasive Vibrio parahaemolyticus infections.

Report	Host factors	Exposure/route	Severe/invasive presentation	Bloodstream involvement	Treatment/intervention	Outcome	Source
Roland, 1970	40 M; none reported	Clamming/bathing in Narragansett Bay	Hemorrhagic rash, shock, leg gangrene	Not specified	Amputation	Survived	Xu et al. [11]
Dobroszycki et al., 1992	48 M; hematologic malignancy/neutropenia	Finger injured by squid	Septicemia with painful swollen finger	Yes	Antimicrobial therapy	Survived	Xu et al. [11]
Rabinowitch et al., 1993	22 M; acute myeloid leukemia	Water-skiing, Patuxent River	Wound infection with septicemia	Yes	Ceftazidime/ciprofloxacin reported	Survived	Xu et al. [11]
Hally et al., 1995	31 F; HCV, cirrhosis, cardiac disease	Oysters and prawns	Septicemia with bullous skin lesions and organ dysfunction	Yes	Ceftriaxone reported	Died	Xu et al. [11]
Ng et al., 1999	60 M; chronic HCV, adrenal insufficiency	Seafood ingestion	Bacteremia, neck/lower-leg SSTI, multiorgan failure	Yes	Ceftazidime + doxycycline; debridement	Survived	Ng et al. [16]
Ralph & Currie, 2007	38 M; diabetes, CHF, atrial fibrillation, chronic airway disease	Fishing/marine exposure	Necrotizing fasciitis, shock, multiorgan dysfunction	Severe systemic infection	Fasciotomy/debridement	Died	Xu et al. [11]
Payinda, 2008	79 M; atrial fibrillation, polymyalgia rheumatica	Leg abraded by boat anchor while fishing	Necrotizing fasciitis, fulminant sepsis	Systemic sepsis	Piperacillin-tazobactam/ciprofloxacin; debridement	Died	Xu et al. [11]
Issack et al., 2008	37 M; alcoholism	Foot injury/sting during marine exposure in Mauritius	Soft-tissue infection	Not specified	Antimicrobials; fasciotomy/debridement	Survived	Xu et al. [11]
Tena et al., 2010	92 F; chronic renal failure, diabetes, malnutrition	Not reported	Fulminant necrotizing fasciitis	Severe systemic infection	Antimicrobial therapy	Died	Xu et al. [11]
Fernando et al., 2011	48 M; CKD, liver transplant, immunosuppression	Fishing in Gulf of Mexico	Septicemia with necrotic skin lesions	Yes	Ceftriaxone + ciprofloxacin; debridement/skin grafting	Survived	Fernando et al. [13]
Ahmad et al., 2013	45 F; diabetes, morbid obesity, chronic HCV	Calf laceration during jet-skiing in brackish water	Necrotizing fasciitis, distributive shock	Yes	Broad-spectrum antimicrobials; fasciotomy	Died	Ahmad et al. [12]
Guillod et al., 2019	85 M; chronic leg ulcers, hepatitis B/right-sided heart failure	Seawater exposure of leg ulcers	Cellulitis, septicemia, septic shock	Yes (4/4 bottles)	Doxycycline + ciprofloxacin; debridement	Survived	Guillod et al. [9]
Chien et al., 2021	71 F; hypertension	Probable seafood ingestion	Small-bowel perforation	Not principal syndrome	Surgery/supportive care	Survived	Chien et al. [8]
Mai et al., 2022	42 M; no major underlying disease reported	Seaside; right-calf abrasion	Bullous septic cellulitis, shock, multiorgan dysfunction/cardiac injury	Septic systemic infection	Antimicrobials; debridement	Survived	Xu et al. [11]
Xu et al., 2024	46 M; HBV, diabetes, IgA nephropathy, hypertension	Market sewage splashed onto chronic foot/calf wound	Necrotizing SSTI, septic shock, multiorgan dysfunction	*V. parahaemolyticus* detected in blood	Broad-spectrum antimicrobials; above-knee amputation	Died	Xu et al. [11]
Junger & Mucelini, 2026	68 M; comorbidities not detailed in abstract	Traumatic foot wound; subsequent coastal-lagoon exposure	Cellulitis, bacteremia, refractory septic shock	Yes	Vancomycin/meropenem → ciprofloxacin/azithromycin	Died	Junger & Mucelini [17]
Aziz et al., 2026	62 M; immunocompetent	Seafood ingestion	Gastroenteritis → bacteremia with TTP-like thrombotic microangiopathy	Yes	Ciprofloxacin + plasma exchange	Survived	Aziz et al. [18]
Present case	Early 60 s M; decompensated alcohol-associated cirrhosis	Occupational raw-fish handling with recurrent skin lacerations; no reported ingestion	Bacteremia with culture-negative neutrocytic ascites/SBP and septic shock	Yes (4/4 bottles)	Ceftriaxone + doxycycline; intensive organ support	Died	Present report

Abbreviations: AF, atrial fibrillation; CHF, congestive heart failure; CKD, chronic kidney disease; HCV, hepatitis C virus; HBV, hepatitis B virus; SBP, spontaneous bacterial peritonitis; SSTI, skin and soft-tissue infection; TTP, thrombotic thrombocytopenic purpura; NR, not reported.

Both gastrointestinal and non-ingestive routes of acquisition have been reported. Severe infections have followed seafood ingestion as well as inoculation of disrupted skin during fishing or exposure to seawater, brackish water, and other potentially contaminated water [9], [11], [12], [13], [16], [17]. Chronic liver disease, diabetes, renal disease, transplantation, hematologic malignancy, and other forms of immunocompromise are frequently represented among reported cases, although severe disease can also occur in patients without recognized immunocompromise [11], [13], [16], [18]. Earlier bacteremia literature likewise demonstrates the longstanding recognition of invasive disease: Ng et al. described bacteremia with soft-tissue infection and multiorgan failure and identified 11 previously reported bacteremic cases [16].

The cases summarized in Table 1 further illustrate the potential severity and heterogeneity of invasive *V. parahaemolyticus* infection. Fatal outcomes have occurred with septicemia, necrotizing soft-tissue infection, and refractory septic shock, whereas survival has been reported following antimicrobial therapy with or without surgical intervention [9], [11], [12], [13], [16], [17], [18]. These case reports should not be used to estimate population-level mortality because they represent a heterogeneous, selectively published group and are subject to publication and selection bias. Nevertheless, they reinforce the importance of considering both seafood ingestion and environmental or occupational marine exposure when evaluating severe infection, particularly in patients with chronic liver disease or other major comorbidities.

Our patient's recurrent skin lacerations while cleaning raw fish provide a plausible non-ingestive route of acquisition. No active wound infection was present when he was evaluated, however, and no wound or environmental cultures were available. The proposed cutaneous route therefore remains inferential. Asymptomatic gastrointestinal acquisition also cannot be completely excluded.

The concurrent neutrocytic ascites requires similar caution. SBP is diagnosed by an ascitic polymorphonuclear cell count ≥ 250 cells/mm³ regardless of culture positivity [14], [15], and culture-negative neutrocytic ascites is generally managed as SBP when the clinical context is appropriate. Our patient's ascitic neutrophil count exceeded 10,000 cells/µL. However, *V. parahaemolyticus* was isolated only from blood and not from ascitic fluid. Prior antibiotic exposure may have reduced ascitic culture sensitivity. Alternatively, the patient may have had culture-negative SBP related to advanced cirrhosis while simultaneously experiencing *V. parahaemolyticus* bacteremia from an extra-abdominal source. Hematogenous peritoneal seeding is biologically plausible but remains speculative without organism recovery from ascitic fluid.

Accordingly, the most defensible interpretation is confirmed *V. parahaemolyticus* bacteremia with concurrent culture-negative neutrocytic ascites/SBP rather than microbiologically confirmed *V. parahaemolyticus* SBP. This distinction avoids over-attribution of the peritoneal process to an organism that was not isolated from that compartment.

Advanced cirrhosis likely contributed substantially to the severity of illness. Cirrhosis is associated with immune dysfunction, impaired bacterial clearance, altered intestinal permeability, and susceptibility to bacterial translocation [14], [15]. These abnormalities may permit relatively minor environmental exposures to progress to severe systemic infection.

Evidence guiding treatment specifically for invasive *V. parahaemolyticus* infection is limited. Doxycycline, fluoroquinolones, and extended-spectrum cephalosporins have activity against Vibrio species, while much of the evidence supporting combination therapy for severe septicemia is derived from *V. vulnificus* rather than *V. parahaemolyticus* [19]. In this case, bacteremia cleared and ascitic neutrophilia improved after cephalosporin and doxycycline therapy, although the patient's overall course remained poor because of advanced liver failure, renal failure, persistent shock, gastrointestinal complications, and subsequent *C. glabrata* fungemia.

This case also has preventive implications. Patients with advanced liver disease should be counseled to avoid raw or undercooked seafood and to protect open wounds from seawater and raw seafood exposure. For occupationally exposed individuals, gloves and appropriate wound protection should be emphasized when handling raw marine products.

Environmental conditions may also influence *V. parahaemolyticus* epidemiology. Sea-surface temperature is a major determinant of *V. parahaemolyticus* abundance, and infections in temperate regions tend to increase during warmer months [20]. Climate anomalies associated with unusually warm coastal waters have been linked to geographic and seasonal expansion of *V. parahaemolyticus* illness risk [20]. These ecological observations support heightened clinical awareness during warm-water periods, but individual case reports cannot establish that climate trends caused a specific infection. Because month of exposure was inconsistently reported across the historical case reports in Table 1, we did not perform a case-level seasonality analysis.

The principal limitations are the absence of *V. parahaemolyticus* isolation from ascitic fluid, the lack of microbiologic confirmation of a cutaneous portal of entry, and multiple competing causes of systemic inflammation and organ failure. Nevertheless, the combination of microbiologically confirmed bacteremia, a detailed occupational exposure history, and concurrent marked neutrocytic ascites highlights an unusual presentation relevant to clinicians caring for patients with advanced liver disease.

## Conclusion

Invasive *V. parahaemolyticus* infection should be considered in patients with advanced liver disease who develop Gram-negative bacteremia or severe sepsis and have marine, seafood-handling, or wound exposure, even without raw seafood ingestion. In cirrhotic patients with neutrocytic ascites, bloodstream infection with an unusual marine pathogen may occur concurrently with culture-negative SBP, but direct peritoneal infection should not be assumed without recovery of the organism from ascitic fluid. A detailed occupational and environmental exposure history can be critical to recognition, treatment, and prevention.

## CRediT authorship contribution statement

**Thi Vy Ngoc Truong:** Writing – original draft, Investigation, Formal analysis, Conceptualization. **Hac-Tu T. Chung:** Writing – review & editing, Project administration, Investigation. **Sai Pavan Kumar Chintalapati:** Writing – review & editing, Validation, Investigation. **Pooja Gurram:** Writing – review & editing, Validation, Supervision, Formal analysis, Conceptualization.

## Consent for publication

Written informed consent for publication of this case report was obtained from the patient. Additional written consent was obtained from the patient's sister. Copies of the written consent forms are available for review by the Editor-in-Chief upon request.

## Ethics declaration

Written informed consent to take part in the study and to publish the article has been obtained from all participants or their legal representatives. The privacy rights of participants have been observed.

This study was performed in compliance with relevant laws, regulatory frameworks and guidelines where the research took place. Ethics committee approval was not required under relevant laws and institutional guidelines. Ethics committee approval was not required for this single-patient case report because it does not constitute human subjects research under applicable institutional guidelines. Written informed consent for publication was obtained from the patient, with additional written consent obtained from the patient's sister.

This research follows the CARE guidelines and the CARE checklist.

## Ethics approval

Institutional Review Board approval was not required because this report describes a single clinical case and does not constitute human subjects research.

## Funding

The authors received no specific funding for this work.

## Declaration of Competing Interest

The authors declare that they have no known competing financial interests or personal relationships that could have appeared to influence the work reported in this paper.

## Data Availability

All relevant clinical information is contained within this case report. Additional information is not publicly available because of patient privacy considerations.
